# The Multifaceted Role of the *FLNC* Gene in Hereditary Cardiomyopathies and Prognosis

**DOI:** 10.3390/genes17091136

**Published:** 2026-09-17

**Authors:** Maria D’Apolito, Alessandra Ranaldi, Teresa Gaudiano, Angela Bruna Maffione, Rosa Santacroce, Giovanna D’Andrea, Massimo Iacoviello, Maurizio Margaglione

**Affiliations:** 1 Medical Genetics, Department of Clinical and Experimental Medicine, University of Foggia, 71122 Foggia, Italy; maria.dapolito@unifg.it (M.D.); alessandra.ranaldi@unifg.it (A.R.); teresa_gaudiano.616418@unifg.it (T.G.); giovanna.dandrea@unifg.it (G.D.); 2 Human Anatomy, Department of Clinical and Experimental Medicine, University of Foggia, 71122 Foggia, Italy; angelabruna.maffione@unifg.it; 3 Cardiology Unit, Polyclinic University Hospital, Viale L. Pinto 1, 71100 Foggia, Italy; massimo.iacoviello@unifg.it

**Keywords:** *FLNC*, DCM, ACM, HCM, RCM

## Abstract

Variants in the filamin C (*FLNC*) gene are increasingly recognized as causes of cardiac and skeletal muscle disease. Cardiomyopathy-associated *FLNC* variants span dilated cardiomyopathy (DCM), nondilated left ventricular cardiomyopathy (NDLVC), arrhythmogenic cardiomyopathy (ACM), hypertrophic cardiomyopathy (HCM), and restrictive cardiomyopathy (RCM), but their clinical interpretation depends strongly on variant class and evidence of pathogenicity. *FLNC* truncating variants (*FLNC*tv), including nonsense, frameshift, and canonical splice-site variants, usually cause haploinsufficiency through nonsense-mediated decay and are predominantly associated with DCM/NDLVC and left-dominant ACM. These phenotypes frequently show subepicardial or circumferential ring-like fibrosis and clinically important ventricular arrhythmias that may occur despite only mild left ventricular dysfunction. On the other hand, selected pathogenic missense variants, particularly within the actin-binding domain or ROD2 immunoglobulin-like repeats 18–21, may promote protein misfolding and aggregation and have been associated with HCM or RCM, including the distinctive saw-tooth myocardial phenotype. However, most reported *FLNC* missense variants remain variants of uncertain significance and must not be considered disease-causing on the basis of location alone. This narrative review summarizes FLNC molecular biology, genotype-phenotype relationships, imaging features, arrhythmic risk, and implications for clinical management. It also discusses the limited evidence linking *FLNC*tv to arrhythmogenic mitral valve prolapse with mitral annular disjunction. Accurate variant classification and phenotype integration are essential for diagnosis and for individualized prevention of sudden cardiac death (SCD).

## 1. Introduction

Filamin C (FLNC) is a large structural protein expressed in the striated skeletal and cardiac muscle, where it plays an essential role in muscle development, force transmission, and the structural maintenance of muscle fibres during contraction. FLNC is encoded by the *FLNC* gene, located on chromosome 7q32.1, which encompasses over 29.5 kb of genomic DNA and comprises 48 coding exons in the canonical transcript (ENST00000325888.13/NM_001458.5) [1].

Filamin C is a 290.8 kDa protein (NP_001449.3) composed of 2725 amino acids with a highly modular architecture that reflects its different roles [2]. FLNC is most prevalent in skeletal and cardiac muscles, where it contributes to structural integrity by localizing to Z-discs, myotendinous junctions, the sarcolemma, and intercalated discs to maintain muscle fibre structure during contraction [3]. This protein acts as a molecular scaffold, cross-linking actin filaments and modulating signalling pathways to coordinate muscle function and repair, thereby enhancing sarcomere stability. The C-terminal immunoglobulin-like repeat 24 mediates homodimerization, allowing the formation of functional crosslinks [4]. The FLNC protein consists of an amino-terminal spectrin-related actin-binding domain spanning amino acids 1–259, which mediates the protein’s attachment to actin filaments, with two calponin-homology domains: CH1 (amino acids 36–142) and CH2 (amino acids 159–262). These tandem CH domains fold together to create the functional actin-binding site, with precise spacing and orientation critical for effective filament recognition and crosslinking. The main role of these domains is to maintain the structural integrity of the sarcomere through crosslinking of actin filaments and anchoring of sarcolemmal proteins to the cytoskeleton [5].

**Figure 1:**
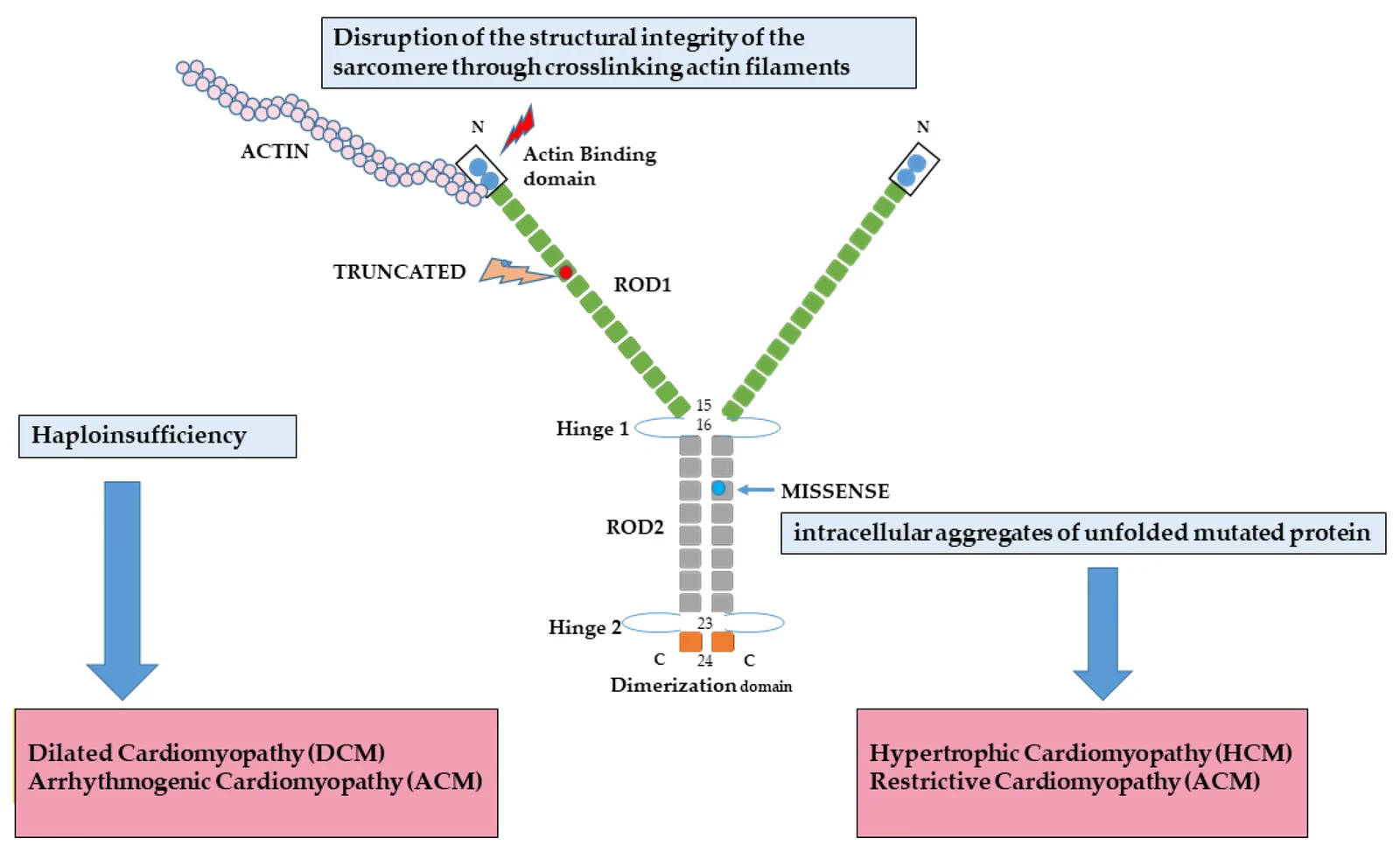
Filamin C and Cardiomyopathies: different phenotypes of cardiomyopathy associated with *FLNC* variants. Protein structure encoded by the *FLNC* gene and its respective domains: the N-terminal portions of filamin C contain Actin Binding Domains (blue); the ROD1 domain (Ig 1–15, green) and the ROD2 domain (Ig 16–23, grey); flexible hinge regions situated between domains 15 and 16 (hinge 1) and between domains 23 and 24 (hinge 2); the dimerization domain Ig-like 24 (orange).

The N-terminal actin-binding domain of filamin C is followed by a rod-like region comprising 24 immunoglobulin-like (Ig-like) domains. These are linked by flexible hinge regions situated between domains 15 and 16 (hinge 1) and between domains 23 and 24 (hinge 2). The Ig-like domains are grouped into ROD1 domain (Ig 1–15) and ROD2 domain (Ig 16–23), which exhibit distinct ligand-binding properties due to their structural organization. The ROD1 domain is more stretched and lacks interdomain interactions in contrast to the ROD2 domain, which is more compact and globularly arranged by domain pairs. These organizational differences between the domains explain why certain ligands bind exclusively to ROD1 or ROD2 [5] (Figure 1).

Homodimerization is mediated by the C-terminal immunoglobulin-like repeat 24 and is required for effective actin cross-linking. The repeated domains also provide binding sites for a series of proteins, allowing filamin C to integrate cytoskeletal organization, mechanotransduction, and cellular signaling.

In cardiac muscle, filamin C localizes to the Z-disc and intercalated discs, regions where individual cardiomyocytes connect and coordinate their contractile activity. In skeletal muscle, the protein is similarly concentrated at Z-discs and plays a role in maintaining muscle fibre integrity during forceful contractions. A series of evidence suggests that filamin C also participates in muscle repair and regeneration following injury, helping to restore normal tissue architecture [5].

## 2. Methods

This narrative review was informed by searches of PubMed/MEDLINE from database inception to 17 July 2026. Search terms combined ‘FLNC’ or ‘filamin C’ with ‘cardiomyopathy’, ‘dilated cardiomyopathy’, ‘nondilated left ventricular cardiomyopathy’, ‘arrhythmogenic cardiomyopathy’, ‘hypertrophic cardiomyopathy’, ‘restrictive cardiomyopathy’, ‘ventricular arrhythmia’, ‘ventricular fibrillation’, ‘sudden cardiac death’, ‘mitral valve prolapse’, and ‘mitral annular disjunction’. Reference lists of key original studies, reviews, and professional guidelines were also screened.

We prioritized peer-reviewed human studies reporting genotype-phenotype associations, imaging findings, arrhythmic outcomes, or functional evidence relevant to variant mechanism. Foundational experimental studies and current professional guidelines were included when they clarified biological mechanisms or clinical management. Reports without sufficient genetic or phenotypic detail and publications not directly relevant to FLNC-related cardiac disease were excluded. Because the aim was a clinically oriented narrative synthesis rather than a systematic review or meta-analysis, study selection was based on relevance and evidentiary contribution; no formal risk-of-bias scoring or quantitative evidence synthesis was performed.

## 3. Genetic Characteristics of FLNC Variants

Pathogenic variants in the *FLNC* gene disrupt the normal architecture and function of muscle tissue and are associated with a wide range of clinical phenotypes, from inherited cardiomyopathies to skeletal muscle diseases [6]. These conditions vary widely in their clinical presentation, from progressive muscle weakness to life-threatening cardiac arrhythmias and heart failure.

Most genetic variants are described in cardiomyopathy patients, as this clinical entity is more prevalent and studies using high-throughput sequencing of *FLNC* are more frequent in cardiomyopathy cohorts.

FLNC-related disorders follow an autosomal dominant inheritance pattern. Although FLNC was traditionally associated with dominant myopathies and cardiomyopathies, patients harboring biallelic *FLNC* variants have recently been documented in cases of isolated disease resulting in more severe, early-onset phenotypes. However, these autosomal recessive patterns are not yet formally recognized in the OMIM or ClinGen databases. The interpretation of these variants remains complex, as it requires integrating population frequency data, computational pathogenicity predictions, and clinical family history [7].

Furthermore, as the *FLNC* gene shows high intolerance to loss-of-function variants, haploinsufficiency is hypothesized to be a primary driver of the disease phenotype in many affected individuals. Pathogenic *FLNC* variants are primarily categorized into truncating and missense mutations. Although both types induce severe functional protein impairments, they correlate with distinct clinical phenotypes, ranging from inherited cardiomyopathies to myopathies. Truncating mutations, including nonsense variants and frameshift changes, typically result in loss of the C-terminal protein-interaction domains and are predominantly linked to arrhythmogenic and dilated cardiomyopathies. The principal molecular mechanism involves disrupted cytoskeletal organization and the induction of cardiomyocyte apoptosis, finally resulting in contractile dysfunction and cardiac remodeling [8,9,10,11].

Missense substitutions are the most frequently reported *FLNC* coding variants, but only a small minority currently meet criteria for pathogenicity. In the database snapshot summarized in Table 1, 2968 of 3127 missense variants (94.9%) were classified as variants of uncertain significance (VUS), whereas 70 (2.2%) were classified as pathogenic or likely pathogenic. Therefore, neither the presence of an *FLNC* missense variant nor its location within a recognized functional domain is sufficient to establish causality. Interpretation should integrate population frequency, segregation, phenotype specificity, validated functional evidence, computational data, and independent case-level observations according to ACMG/AMP criteria. Domain clustering can support interpretation, particularly for established HCM/RCM-associated regions, but cannot replace variant-level evidence.

It has been suggested that specific variants fall into three pathomechanisms [8]:

1. Variants causing a premature stop codon and concomitant nonsense-mediated decay (NMD), resulting in haploinsufficiency.
2. Variants that are predicted to lead to expression of misfolded proteins, which saturate the proteasome and autophagy pathways.
3. Variants, which give a toxic gain-of-function by altering ligand-binding properties.

*FLNC* variants have predominantly been reported in adult populations with cardiomyopathies, with an average age of onset of 39.7  ±  14.5 years for FLNC-DCM and a mean age of onset of 35.9  ±  14.8 years for HCM in *FLNC* carriers [9,12,13]. *FLNC* variants have been identified in 2.8% of Italian pediatric patients with cardiomyopathies, arrhythmias, and congenital heart defects (CHDs) [14].

The clinical consequences of *FLNC* variants depend on the location and nature of the molecular change. Some variants appear to act through dominant-negative mechanisms, where the altered protein interferes with the function of the normal protein produced from the unaffected gene copy. Other variants may cause disease through haploinsufficiency, where a single functional gene copy cannot produce sufficient protein to maintain normal muscle structure and function [6]. The specific clinical outcome often depends on the variant location, which is associated with specific cardiac or skeletal muscle phenotypes, though significant clinical variability occurs even among individuals carrying the same pathogenic variant.

Potential founder effects have been observed in certain populations, where specific *FLNC* variants occur at higher frequencies due to shared ancestry. The Dutch founder variant (c.6864_6867dup/p.Val2290Argfs*23), tracing back to the southern Netherlands, is a frameshift variant that predisposes carriers to heart failure and ventricular arrhythmias, often presenting with a characteristic ring-like late gadolinium enhancement (LGE) on cardiac MRI [15]. The Hong Kong Chinese founder variant (c.8129G>A/p.Trp2710*), estimated to have arisen 42 to 71 generations ago, underlies a higher regional incidence of FLNC-related myofibrillar myopathy [16]. The Ashkenazi Jewish founder variant (c.3791-1G>C) is a splice-site variant tied to haploinsufficiency, recurrently identified in families with arrhythmogenic cardiomyopathy [17,18].

Additional regional clustering and shared haplotypes have been reported in German and Italian cohorts, pointing to localized founder events driving dilated and arrhythmogenic cardiomyopathies [19].

As summarized in Table 1, variants were normalized to HGVS nomenclature using the canonical *FLNC* transcript NM_001458.5. Records were collated from ClinVar (as accessed on 16 of July, 2026), UniProt (UniProt: release 2026_03), LOVD (LOVD: v3.0 Build 30b), VarSome (VarSome: v13.18.2.0) and variants reported in the peer-reviewed literature, with the search ending on 17 July 2026.

Exact duplicates were identified by genomic and coding coordinates and counted once. When classifications differed among sources, the ClinVar aggregate classification was used when available; otherwise, the most recent expert-curated classification supported by explicit ACMG/AMP evidence was retained. Conflicting or insufficiently resolved records were assigned to the VUS category rather than upgraded. Coding consequences were grouped as shown in Table 1. Counts are descriptive of a time-dependent database snapshot and do not estimate disease prevalence or penetrance.

MitoMap, which catalogs mitochondrial DNA variation, was removed from the final source list because FLNC is a nuclear gene and it did not provide an appropriate independent source for this analysis. Therefore, the table represents a transparent summary of reported *FLNC* records and not a clinically validated set of disease-causing variants.

## 4. FLNC and Cardiomyopathy Phenotypes

Cardiomyopathies represent disorders of the myocardium defined by structural and functional cardiac abnormalities that cannot be explained by obvious causes like hypertension, coronary artery disease, congenital heart defects, or valvular disease.

According to the most recent European Society of Cardiology guidelines, these conditions are categorized into distinct phenotypes: hypertrophic cardiomyopathy (HCM), dilated cardiomyopathy (DCM), nondilated left ventricular cardiomyopathy (NDLVC), arrhythmogenic right ventricular cardiomyopathy (ARVC), and restrictive cardiomyopathy (RCM) [20].

## 5. FLNC and Dilated Cardiomyopathy (DCM)

Within this classification, dilated cardiomyopathy (DCM) represents a primary driver of heart failure (HF) and constitutes the leading indication for orthotopic cardiac transplantation [21]. The hallmark features of this specific condition include ventricular dilation paired with compromised systolic function in one or both ventricles, occurring in the absence of abnormal loading conditions and severe coronary artery disease. Approximately 35% of all DCM cases are attributable to genetic mutations [21,22,23]. Clinically, familial DCM is diagnosed when there is either (a) a minimum of two relatives affected by DCM, or (b) a single relative diagnosed with DCM alongside a family history of sudden cardiac death (SCD) occurring before the age of 35.

Consequently, the field of molecular genetics is garnering rapidly increasing attention regarding its clinical relevance to DCM. Researchers have identified numerous susceptibility genes capable of inducing phenotypically impactful cardiomyopathies. Among these discoveries, genetic alterations in structural proteins, particularly filamins, are gaining widespread recognition as major causative factors [23,24].

## 6. Pathogenic Profile and Arrhythmic Risk of *FLNC* Truncating Variants in DCM

Mutations in the *FLNC* gene are linked to myocardial disorders that manifest through a variety of cardiomyopathy phenotypes. Such mutations carry an aggressive clinical prognosis, characterized by an elevated risk of ventricular arrhythmias and SCD [25,26]. Consequently, early identification of these variants and the prompt initiation of therapeutic management are vital strategies to mitigate adverse clinical outcomes. Indeed, reducing the incidence of SCD represents the primary objective during the clinical management of patients with DCM.

To improve risk stratification, the Filamin C Registry Consortium recently introduced a five-variable risk model for predicting SCD or major ventricular arrhythmias (MVA) in carriers of *FLNC* truncating variants (*FLNC*tv). This predictive model includes age, male sex, a documented medical history of syncope, nonsustained ventricular tachycardia (NSVT), and left ventricular ejection fraction (LVEF). Although the correlation linking LVEF to arrhythmic risk follows a non-linear pattern, LVEF measurements exceeding 58% correspond to a markedly reduced level of risk [26].

The pathogenic *FLNC* variants associated with DCM consist predominantly of truncating variants (*FLNC*tv) [10], including nonsense, frameshift, and splice-site mutations, classified as (likely) pathogenic, as *FLNC* gene is highly intolerant for loss-of-function variants (pLI-score = 1). In *FLNC*-associated DCM, nonsense-mediated decay (NMD) and subsequent haploinsufficiency were validated as the pathomechanism for a number of truncating variants. Immunohistochemical analysis showed normal FLNC protein in the intercalated discs of patients with DCM, with no detectable abnormal FLNC protein aggregates in the cytoplasm [10]. The absence of aggregates in the cardiac tissue of patients with truncating *FLNC* variants in the ROD2 domain indicates the lack of an abnormal FLNC protein.

These variants generally drive pathogenesis via haploinsufficiency (resulting in a loss of functional protein) rather than toxic protein aggregation, which is instead more characteristic of certain skeletal myopathy variants; this mechanism likely drives fibrotic remodelling.

From a clinical point of view, individuals with *FLNC*tv typically display non-ischemic left ventricular systolic dysfunction accompanied by only subtle left ventricular dilatation [8]. On cardiac magnetic resonance (CMR) imaging, these patients characteristically exhibit widespread mid-wall, subepicardial fibrosis or a circumferential, “ring-like” late gadolinium enhancement (LGE) pattern, alongside a high frequency of ventricular ectopy or NSVT [27,28]. In a subset of cases, sustained ventricular tachycardia/ventricular fibrillation (VT/VF) or SCD can even represent the initial clinical presentation of the disease [28].

Phenotype-positive carriers of *FLNC*tv have a substantial risk of sustained ventricular tachycardia, ventricular fibrillation (VF), sudden cardiac death, or appropriate implantable cardioverter-defibrillator therapy across DCM, NDLVC, and predominantly left-dominant or biventricular ACM presentations. Importantly, this risk is not confined to patients with severely reduced LVEF. Recent *FLNC*tv-specific analyses support genotype-informed risk assessment using age, sex, syncope, NSVT, and LVEF, although the available evidence does not justify describing FLNC as an independent VF predictor specifically in classical right-dominant ARVC [10,25,26,28,29,30,31].

Accordingly, the 2023 European Society of Cardiology (ESC) Guidelines for the management of cardiomyopathies support clinicians in the shared decision-making process for prophylactic cardioverter-defibrillator (ICD) implantation as primary prevention of SCD in patients with dilated cardiomyopathy who present with an LVEF ≥ 35% and an *FLNC* truncating variant genotype, when in the presence of additional risk factors for SCD [20].

## 7. FLNC and Arrhythmogenic Cardiomyopathy (ACM)

Arrhythmogenic cardiomyopathy (ACM) is an inherited, progressive myocardial disorder that predisposes affected individuals to life-threatening ventricular arrhythmias (VA), heart failure (HF), and sudden cardiac death (SCD) [32]. The pathological hallmark of ACM is the progressive, time-dependent replacement of the ventricular myocardium with fibrofatty tissue [33,34]. Approximately 50% of ACM patients carry likely pathogenic or pathogenic (LP/P) variants in genes encoding desmosomal proteins; consequently, ACM is fundamentally classified as a ‘disease of the desmosomes’. These patients typically manifest the right-dominant ACM subtype, known as arrhythmogenic right ventricular cardiomyopathy (ARVC). However, mutations in non-desmosomal genes have also been implicated in ACM pathogenesis [35]. LP/P variants in non-desmosomal genes such as Transmembrane Protein 43 (TMEM43) [36], Phospholamban (PLN) [37], and Desmin (DES) [38] are causative of ARVC. Overall, variants in both desmosomal and non-desmosomal genes contribute to left-dominant (i.e., arrhythmogenic left ventricular cardiomyopathy [ALVC]), right-dominant (ARVC), or biventricular ACM phenotypes [39]. Among these non-desmosomal alterations, mutations in the *FLNC* gene are specifically involved in the pathogenesis of a distinctive left ventricular (LV) phenotype. This FLNC-associated phenotype is characterized by extensive, non-ischemic LV fibrosis (commonly evidenced by cardiac magnetic resonance [CMR] in 75% of cases), life-threatening VAs, and a high incidence of SCD [10,18,25,29]. Notably, SCD events are frequently reported in FLNC cohort studies. Within DCM and ACM populations, SCD presents as the initial symptom in 5% of cases and occurs during follow-up in 15% of individuals [28]. While right-dominant forms have been documented, the left-dominant phenotype represents the most prevalent manifestation, identified in over half of FLNC-associated ACM patients [28]. This specific presentation correlates with the physiological role of the encoded protein, which acts as a structural actin cross-linker between the sarcomeric Z-disc and the sarcolemma, thereby generating a phenotypic overlap between dilated cardiomyopathy (DCM) and ACM [25,29].

## 8. FLNC and Hypertrophic Cardiomyopathy (HCM)

Hypertrophic cardiomyopathy (HCM) is a prevalent, genetically determined myocardial disease characterized by left ventricular (LV) hypertrophy that cannot be solely explained by concomitant cardiac, systemic, or metabolic pathologies. The majority of cases arise from pathogenic variants in genes encoding sarcomeric proteins. A large number of genetic studies have established that HCM is caused mainly by mutations in 11 or more genes encoding sarcomere components: thick (myosin) and thin myofilament protein, adjacent Z-band or M band, calcium homeostasis, or sarcomere-associated proteins, which are expressed primarily or exclusively in the heart [40].

Recent molecular surveillance has implicated non-sarcomeric genes, such as FLNC (encoding filamin C), in the pathogenesis and clinical phenotypic variation in HCM [24].

HCM predominantly exhibits an autosomal dominant inheritance pattern. Missense variants in *FLNC* constitute approximately 1.3% to 8.7% of HCM cohorts, representing a minor yet clinically significant fraction (~2%) of variant-positive cases [41,42,43]. Mechanistically, these missense substitutions alter the three-dimensional tertiary structure of filamin C, disrupting its physiologic interactions with key cytoskeletal elements and compromising cell signaling pathways. Structural topology analysis indicates a critical clustering of pathogenic missense variants within the ROD2 domain, a region essential for cellular signaling. Missense mutations localized to the ROD2 domain display a high propensity to form intracellular protein aggregates, which trigger cellular stress responses, specifically upregulating autophagy and the ubiquitin-proteasome pathway, that drive myofibrillar degeneration and severe myofibrillar disarray. Ex vivo histological evaluations of myocardium harboring *FLNC* variants demonstrate distinct abnormalities, including profound myofibrillar disarray, interstitial fibrosis, and dense sarcomeric aggregates [44]. Notably, these specific aggregative pathologies are absent in healthy controls and in HCM patients with wild-type FLNC [41]. FLNC-related cardiomyopathy patients rarely display skeletal myopathy. Patients affected by *FLNC* missense variants show a higher risk of SCD than patients with HCM without pathogenic variants; although *FLNC* variants account for only 2% of variant-positive HCM cases, the affected individuals frequently present severe phenotypes and clinical outcomes [44].

## 9. FLNC and Restrictive Cardiomyopathy (RCM)

Restrictive cardiomyopathy (RCM) represents a rare and heterogeneous group of myocardial diseases, constituting the least common form of cardiomyopathy. The condition is characterized by a marked increase in myocardial stiffness, which leads to impaired ventricular filling, elevated end-diastolic pressures, and subsequent atrial enlargement [45]. From a biological point of view, RCM is defined by severe ventricular diastolic dysfunction with initially preserved or near-normal systolic function, alongside a relatively normal left ventricular (LV) volume. While systolic function remains preserved early in the disease course, significant systolic impairment typically manifests only during end-stage disease [45].

Diagnosing RCM remains clinically challenging due to its varied pathogenesis, diverse clinical presentations, and complex diagnostic evaluation. A key diagnostic dilemma involves differentiating RCM from hypertrophic cardiomyopathy (HCM) [46]. Mild left ventricular hypertrophy (LVH) may be present in RCM patients, mimicking HCM features. Conversely, individuals with primary HCM can exhibit “restrictive physiology” characterized by impaired ventricular filling and atrial dilation, though typically to a lesser degree than observed in pure RCM. Notably, the previous literature indicates that restrictive forms of HCM are an uncommon subtype, accounting for approximately 1.5% of all HCM cases [46].

Genetic analyses have identified several candidate pathogenic gene variants associated with RCM, including *TNNI3, TNNT2, MYL2, FLNC*, and *MYH7*. Among these, *FLNC* variants associated with a spectrum of filaminopathies have been linked to specific phenotypes including supraventricular arrhythmias, a high prevalence of atrial fibrillation, atrial cardiomyopathies, diastolic dysfunction, and variable degrees of skeletal or myofibrillar myopathy [47,48,49,50,51].

A review of previously identified *FLNC* missense variants in RCM patients highlights a mutational hotspot localized within the immunoglobulin-like (Ig-like) repeat domains 18 to 21 (d18-21) of the ROD2 domain. This specific region is critical for FLNC dimerization, secondary protein structure acquisition, and interactions with Z-disc, muscle development, and contraction-related proteins, as well as serving as a crucial site for protein phosphorylation. Rare missense variants within this ROD2 subdomain can precipitate protein misfolding and impaired crosslinking, ultimately leading to sarcomeric disarray and mechanotransduction impairment. Phenotypically, these d18-21 variants manifest as a unique “saw-tooth myocardium,” a severe mixed HCM/RCM phenotype characterized by unusual saw-tooth LV hypertrophy, deep myocardial recesses, heart failure (HF) symptoms, and profound diastolic dysfunction [52]. RCM presents unique clinical management challenges due to its relentlessly progressive nature and the paucity of effective treatment options. In pediatric cohorts, a pathogenic or likely pathogenic genetic variant is identified in approximately 50% of cases. Symptoms of heart failure usually develop at a young age in these pediatric patients, frequently necessitating early heart transplantation [50].

The genotype–phenotype relationships, proposed pathogenic mechanisms, imaging findings, and arrhythmic risk associated with the main classes of FLNC variants are summarized in Table 2.

## 10. Mitral Valve Prolapse and Mitral Annular Disjunction

Mitral annular disjunction (MAD) is a structural separation between the posterior mitral annulus and the adjacent ventricular myocardium and is frequently assessed in the setting of myxomatous or bileaflet mitral valve prolapse (MVP). MAD and the associated mechanical stretch of the inferobasal myocardium and papillary muscles may contribute to fibrosis and ventricular ectopy. However, MAD is a phenotypic imaging feature rather than an established FLNC-specific cardiomyopathy subtype.

A familial report identified the *FLNC* truncating variant p.Trp34* in relatives with bileaflet MVP, MAD, left ventricular enlargement, frequent premature ventricular contractions, and NSVT, suggesting that *FLNC* haploinsufficiency may provide a proarrhythmic myocardial substrate in selected families [53]. This observation is biologically plausible because reduced filamin C may weaken cardiomyocyte adhesion and mechanotransduction, potentially increasing vulnerability to mechanical stress around the mitral apparatus. Nevertheless, evidence is currently limited to a small pedigree and does not prove that FLNC causes MAD or independently predicts VF among patients with MVP/MAD. In clinical practice, an *FLNC* variant found in this setting should be interpreted using standard variant-level criteria and integrated with family segregation, ventricular phenotype, CMR fibrosis, ectopic burden, NSVT, syncope, and ventricular function. Larger genotype-first cohorts are needed before FLNC can be incorporated into MAD-specific risk algorithms.

## 11. Future Perspectives

Future perspectives in arrhythmic risk stratification and the evolution of Next-Generation Sequencing (NGS) technologies are currently redefining the clinical management of patients carrying variants in the *FLNC* gene. The primary objective of contemporary research is to shift from a reactive management strategy traditionally based solely on symptomatology or Left Ventricular Ejection Fraction (LVEF) toward an early, personalized precision medicine approach.

The paramount clinical challenge regarding *FLNC* variants lies in the sharp discrepancy between the severity of ventricular arrhythmias (which pose a high risk of Sudden Cardiac Death, SCD) and the stability of myocardial contractile function, which frequently appears preserved. Consequently, future clinical strategies focus heavily on the implementation of multiparametric models. The systematic clinical application of the 5-variable risk model (incorporating age, male sex, syncope, non-sustained ventricular tachycardia [NSVT], and LVEF) developed by the Filamin C Registry Consortium will establish the benchmark standard for guiding prophylactic implantable cardioverter-defibrillator (ICD) implantation. Advances in Cardiovascular Magnetic Resonance (CMR) will expand beyond the conventional assessment of macroscopic fibrosis to characterize microscopic diffuse fibrosis, thereby intercepting fibrotic remodeling before it becomes clinically manifest.

A major focus of future research aims to establish stringent longitudinal monitoring protocols for genetically positive, phenotype-negative carriers identified through cascade screening, to detect the earliest signs of electrical instability.

To overcome the limitations of standard genetic screening, molecular diagnostics are shifting toward more comprehensive techniques such as transitioning to WES and WGS to map variants within deep intronic or regulatory regions of the *FLNC* gene. This framework elucidates cases with highly suspected familial cardiomyopathy previously classified as negative or associated with variants of uncertain significance (VUS). The introduction of Long-Read Sequencing technologies could facilitate the identification of complex structural rearrangements, large insertions/deletions (indels), or duplications (such as the Dutch founder variant) that typically evade conventional short-read sequencing. The combined application of NGS and RNA sequencing could allow for the functional validation of splicing variants. Finally, in the near future, the clinical impact of a high-penetrance monogenic *FLNC* variant will be contextualized alongside a Polygenic Risk Score (PRS), which maps thousands of modifier variants across the rest of the genome that can either amplify or mitigate individual susceptibility to life-threatening arrhythmias. Taken together, these developments are expected to move the clinical management of FLNC-related disease from a reactive, symptom-driven model toward proactive, genotype-informed risk stratification.

## 12. Conclusions

FLNC-related cardiomyopathy is best understood through the joint interpretation of variant class, variant-level pathogenicity, cardiac phenotype, imaging, and arrhythmic markers. FLNC truncating variants are most consistently associated with DCM/NDLVC and left-dominant ACM with clinically important ventricular arrhythmias, whereas a limited subset of pathogenic missense variants is associated with HCM or RCM. Clinical relevance of most missense variants remains uncertain. Their identification should not guide diagnosis or device therapy without additional evidence. The possible relationship between FLNC haploinsufficiency and arrhythmogenic MVP/MAD is clinically intriguing but remains unproven beyond limited familial observations.

## Figures and Tables

**Table 1:** Descriptive snapshot of 5805 unique *FLNC* variants reported in ClinVar, UniProt, LOVD, VarSome, and the peer-reviewed literature (until 17 July 2026). Records were normalized to NM_001458.5, and duplicates were removed. The totals comprise 712 pathogenic/likely pathogenic variants, 3317 variants of uncertain significance, and 1776 benign/likely benign variants.

Coding Impact	Pathogenic	Likely Pathogenic	Uncertain Significance	Likely Benign	Benign	Total
**Synonymous**	0	0	89	1330	89	1508
**Missense**	18	52	2968	78	11	3127
**Nonsense**	145	27	6	0	0	178
**Stoploss**	0	0	1	0	0	1
**Frameshift**	286	61	11	0	0	358
**Inframe Indel**	1	1	66	0	0	68
**Splice junction loss**	24	96	27	0	0	147
**Non-coding**	1	0	149	251	17	418
**Total**	475	237	3317	1659	117	5805

**Table 2:** Clinically oriented summary of FLNC variant classes, mechanisms, phenotypes, imaging features, and arrhythmic risk. Abbreviations: FLNCtv, FLNC truncating variants; NMD, nonsense-mediated decay; DCM, dilated cardiomyopathy; NDLVC, nondilated left ventricular cardiomyopathy; ACM, arrhythmogenic cardiomyopathy; LGE, late gadolinium enhancement; VT/VF, ventricular tachycardia/ventricular fibrillation; SCD, sudden cardiac death; ICD, implantable cardioverter-defibrillator; ARVC, arrhythmogenic right ventricular cardiomyopathy; HCM, hypertrophic cardiomyopathy; RCM, restrictive cardiomyopathy; MVP, mitral valve prolapse; MAD, mitral annular disjunction; NSVT, nonsustained ventricular tachycardia; VF, ventricular fibrillation; LV, left ventricular.

Variant Type or Domain	Likely Mechanism	Predominant Phenotype	Typical Imaging Findings	Arrhythmic Risk	Interpretive Caution
*FLNC* Truncating variants	NMD and haploinsufficiency	DCM, NDLVC, left-dominant or biventricular ACM	Subepicardial or mid-wall LGE; circumferential ring-like fibrosis; often limited dilation	High risk of VT/VF, SCD, or ICD therapy; events may precede severe LV dysfunction	Apply FLNCtv-specific risk assessment; do not equate all cases with classical right-dominant ARVC
Pathogenic missense variants in actin-binding domain	Altered actin binding; dominant-negative or toxic effect proposed	HCM; occasional overlapping phenotypes	LV hypertrophy and myocardial fibrosis; pattern varies by variant	Not adequately quantified independently of phenotype	Location supports but does not establish pathogenicity
Pathogenic missense variants in ROD2, especially repeats 18–21	Misfolding, aggregation, impaired mechanotransduction	RCM or mixed HCM/RCM	Saw-tooth myocardium, deep recesses, atrial enlargement, severe diastolic dysfunction	Atrial arrhythmias common; ventricular risk remains incompletely defined	Most missense variants are VUS; require variant-level evidence
Rare *FLNC*tv reported with bileaflet MVP and MAD	Possible mechanically amplified arrhythmogenic substrate	Arrhythmogenic MVP/MAD reported in one family	MAD, inferobasal or papillary-muscle fibrosis, LV enlargement	Potential ventricular ectopy/NSVT; independent VF risk unproven	Association is hypothesis-generating, not sufficient for causal or predictive classification

## Data Availability

Not applicable. No new data were created or analyzed in this review; all data discussed are available in the cited publications and public variant databases (UniProt, ClinVar, LOVD, VarSome).

## Abbreviations

The following abbreviations are used in this manuscript:

FLNC	Filamin C (gene/protein)
DCM	Dilated cardiomyopathy
HCM	Hypertrophic cardiomyopathy
ACM	Arrhythmogenic cardiomyopathy
ARVC	Arrhythmogenic right ventricular cardiomyopathy
ALVC	Arrhythmogenic left ventricular cardiomyopathy
NDLVC	Nondilated left ventricular cardiomyopathy
RCM	Restrictive cardiomyopathy
FLNCtv	FLNC truncating variant(s)
NMD	Nonsense-mediated decay
SCD	Sudden cardiac death
MVA	Major ventricular arrhythmia(s)
VA	Ventricular arrhythmia(s)
VT/VF	Ventricular tachycardia/ventricular fibrillation
NSVT	Nonsustained ventricular tachycardia
LVEF	Left ventricular ejection fraction
LVH	Left ventricular hypertrophy
HF	Heart failure
ICD	Implantable cardioverter-defibrillator
CMR	Cardiovascular magnetic resonance
LGE	Late gadolinium enhancement
VUS	Variant(s) of uncertain significance
NGS	Next-generation sequencing
WES	Whole-exome sequencing
WGS	Whole-genome sequencing
PRS	Polygenic risk score
pLI	Probability of loss-of-function intolerance
TMEM43	Transmembrane protein 43
PLN	Phospholamban
DES	Desmin
ESC	European Society of Cardiology
MAD	Mitral annular disjunction
MVP	Mitral valve prolapse
PVC	Premature ventricular contraction
